# Validity and reliability of the medial temporal lobe atrophy scale in a memory clinic population

**DOI:** 10.1186/s12883-021-02325-2

**Published:** 2021-07-24

**Authors:** Anna Molinder, Doerthe Ziegelitz, Stephan E. Maier, Carl Eckerström

**Affiliations:** 1 Department of Radiology, Institute of Clinical Sciences, Sahlgrenska Academy, University of Gothenburg, Gothenburg, Sweden; 2 Neuroradiology, Sahlgrenska sjukhuset, Blå stråket 5, Gothenburg, 413 46 Sweden; 3 Department of Radiology, Brigham and Women’s Hospital, Harvard Medical School, Boston, MA USA; 4 Department of Psychiatry and Neurochemistry, Institute of Neuroscience and Physiology, Sahlgrenska Academy, University of Gothenburg, Gothenburg, Sweden; 5 Department of Immunology and Transfusion Medicine, Region Västra Götaland, Sahlgrenska University Hospital, Gothenburg, Sweden

**Keywords:** Dementia, Medial temporal lobe atrophy (MTA), Alzheimer’s disease, Mild cognitive impairment, Atrophy, Magnetic resonance imaging

## Abstract

**Background:**

Visual rating of medial temporal lobe atrophy (MTA) is often performed in conjunction with dementia workup. Most prior studies involved patients with known or probable Alzheimer’s disease (AD). This study investigated the validity and reliability of MTA in a memory clinic population.

**Methods:**

MTA was rated in 752 MRI examinations, of which 105 were performed in cognitively healthy participants (CH), 184 in participants with subjective cognitive impairment, 249 in subjects with mild cognitive impairment, and 214 in patients with dementia, including AD, subcortical vascular dementia and mixed dementia. Hippocampal volumes, measured manually or using FreeSurfer, were available in the majority of cases. Intra- and interrater reliability was tested using Cohen’s weighted kappa. Correlation between MTA and quantitative hippocampal measurements was ascertained with Spearman’s rank correlation coefficient. Moreover, diagnostic ability of MTA was assessed with receiver operating characteristic (ROC) analysis and suitable, age-dependent MTA thresholds were determined.

**Results:**

Rater agreement was moderate to substantial. MTA correlation with quantitative volumetric methods ranged from -0.20 (*p*< 0.05) to -0.68 (*p* < 0.001) depending on the quantitative method used. Both MTA and FreeSurfer are able to distinguish dementia subgroups from CH. Suggested age-dependent MTA thresholds are 1 for the age group below 75 years and 1.5 for the age group 75 years and older.

**Conclusions:**

MTA can be considered a valid marker of medial temporal lobe atrophy and may thus be valuable in the assessment of patients with cognitive impairment, even in a heterogeneous patient population.

## Background

The medial temporal lobe (MTL) is an early affected site for Alzheimer’s disease (AD) related neurodegeneration [1]. Regional atrophy of the MTL structures detected with magnetic resonance imaging (MRI) is a recognized AD biomarker [2, 3]. However, MTL atrophy may be present in other types of dementia, e.g. in subcortical vascular dementia (SVD) [4–8] and is independently associated with cognitive impairment in patients with cerebral vascular pathology [6, 9, 10]. MTL atrophy may also be present in patients with mild cognitive impairment (MCI) [11]. In this patient group and even in healthy individuals, MTL atrophy or increased atrophy rate indicates risk of future cognitive decline [12–15].

Assessment of MTL atrophy on MRI is often part of the standard evaluation of patients with cognitive decline. There are several (semi-)automated segmentation tools available for quantifying MTL volumes, but the availability and usage of such tools vary across radiological departments. Furthermore, absolute hippocampal volumes will be biased by the quantitative measuring method used, since manual volumetry and the various automated software programs tend to delineate the anatomical structures differently [16]. In terms of easy clinical applicability, visual assessment of MTL atrophy is still superior to volumetric measuring methods. For visual assessment the medial temporal lobe atrophy scale (MTA) introduced by Scheltens et al. is widely used [17, 18]. In the original article, the MTA scale was able to differentiate between AD patients and controls, a finding that has been replicated in later studies [19–21]. Depending on methods used, comparisons between MTA and manual volumetry or automated methods have shown acceptable to good correlations [22–26]. Studies of MTA with regard to reliability, validity and diagnostic ability, however, have mostly focused on AD and its prodromal phases, fewer on SVD or mixed dementia.

The patient population admitted at memory clinics is characterized by rather diverse cognitive symptoms and underlying disorders, sometimes with mixed neurodegenerative and vascular pathology. Such a mixed clinical patient population, ranging from subjective cognitive impairment (SCI) and MCI to dementia including AD, SVD and mixed dementia, is the subject of the present report.

The overall aim of the study was to investigate the reliability and validity of the MTA scale, with regard to both quantitative hippocampal volumes and to clinical diagnoses, using a well-defined memory clinic patient cohort with different underlying disorders and different stages of cognitive impairment.

## Methods

### Study participants

#### The Gothenburg MCI study

The present study is part of the Gothenburg MCI study [27], a clinical longitudinal study focused on neurodegenerative, vascular and stress disorders prior to the development of dementia. The Gothenburg MCI study was approved by the local ethics committee (approval number: L091-99, 1999; T479-11, 2011), and is conducted in accordance with the Declaration of Helsinki of 1975 and 1983. Written informed consent is obtained from all participants in the Gothenburg MCI study.

The study participants for the Gothenburg MCI study were recruited at the Memory Clinic, where they were examined due to subjective or objective cognitive complaints. Inclusion criteria for the Gothenburg MCI study were: age between 50 to 79 years; mini mental state examination (MMSE) score > 18; duration of cognitive decline for 6 or more months. Exclusion criteria consisted of somatic diseases that may cause cognitive impairment, e.g., brain tumors, subdural hemorrhage, encephalitis, unstable heart disease or hypothyroidism as well as severe psychiatric disorders, substance abuse or confusion caused by drugs. Controls were primarily recruited through senior citizen organizations. In a few cases, the controls were spouses to patients at the memory clinic. Additionally, twenty-three patients were reclassified as healthy controls when they upon examination had neither objective nor subjective signs of cognitive impairment. Inclusion and exclusion criteria were the same as for the patients with the exception that controls were not included if they had subjective or objective signs of cognitive disorders.

#### Present study

Participants from the Gothenburg MCI study were included in the present study if they had undergone at least one MRI exam during the observation period, with a technically successful T_1_-weighted volume scan suitable for medial temporal lobe atrophy (MTA) evaluation. Between 1999 and 2014, 458 patients and 73 controls underwent both MRI and clinical examination, including a global deterioration scale (GDS) classification, as part of the Gothenburg MCI study. A total of 756 MRI scans were performed, i.e., some of the enrolled subjects underwent more than one MRI examination. Four of these scans, obtained in four patients who underwent only a single MRI examination, had to be excluded because of distortion artifacts or inadequate volume coverage for MTA assessment. Participants entering the study as patients (*N* = 454) received 655 MRI examinations and controls (*N* = 73) 97 MRI exams. Out of the total of 752 MRI examinations included in the study, 136 were performed with a 0.5 Tesla scanner and 616 were performed with a 1.5 Tesla scanner. Each MRI exam, whether performed at baseline or at follow up, was accompanied by a new clinical assessment including GDS classification. Follow up time ranged from 1 to 9 years.

For the purpose of this study, all included MRI exams were grouped according to the subject’s GDS classification at the time of each MRI scan, regardless as to whether the participant had entered as patient of the Gothenburg MCI study or as presumed healthy control. The cognitively healthy cohort (CH) comprises 105 examinations.

### Clinical evaluation

At each clinic visit, participants were classified according to the GDS, based on anamnestic data and assessment of cognitive symptoms using the following clinical checklists: Stepwise Comparative Status Analysis (STEP); I-Flex, short form of the Executive Interview (EXIT); Mini mental state examination (MMSE); and Clinical Dementia Rating (CDR). GDS 1 stands for cognitively intact, GDS 2 for SCI, GDS 3 for MCI and GDS 4 for mild dementia [28]. The CDR sum of boxes assessment was based on information from both the patient and an informant. The guidelines for the classification were as follows: For GDS 2 (SCI) participants should have MMSE ≥ 28, CDR ≤ 0.5, I‐FLEX < 3, and no positive outcomes on variables 13‐20 of STEP; GDS 3 (MCI) corresponds to MMSE ≥ 26, CDR > 0.5, I‐FLEX ≤ 3, and one or fewer positive outcomes on variables 13‐20 of STEP; and for GDS 4 (mild dementia) participants should have MMSE ≤ 25, CDR > 1.0, STEP > 1, and I‐FLEX > 3. When the guidelines were not applicable, a consensus decision among the physicians at the clinic was made to determine the appropriate GDS score.

The detailed diagnostic procedures and further details concerning the Gothenburg MCI study design have been presented in an earlier publication [27].

Study participants with GDS 4 (dementia) were further classified according to specific diagnoses, with AD (98 MRI exams) according to the NINCDS-ADRDA criteria [29], subcortical vascular dementia (25 MRI exams) according to the Erkinjuntti criteria [30] or mixed Alzheimer/vascular dementia (51 MRI exams). For mixed dementia, AD criteria had to be fulfilled as well as moderate/severe white matter changes (WMC) (Fazekas score ≥ 2) on MRI, or mild WMC in combination with a marked fronto-subcortical-dysexecutive syndrome. The clinician who set the dementia diagnoses had access to MRI images but was blinded to volumetric and visual rating data, as well as neuropsychological test results and cerebrospinal fluid (CSF) biomarker data.

Furthermore, a diagnostically heterogeneous group with GDS 4 was summarized as “Other dementias” and includes: Twenty-one examinations that were performed in participants with dementia *non ultra descripta*, ten with dementia of uncertain etiology, four with fronto-temporal dementia according to Neary et al. [31], two with mixed fronto-temporal dementia and vascular dementia, two with primary progressive aphasia according to Gorno-Tempini et al. [32] and one with Lewy body dementia according to McKeith et al. [33]. These dementia subgroups are not included in analyses concerning classification accuracy, due to their small group sizes. Average demographical and clinical data of respective groups at the time of MRI examination are presented in Table 1.

**Table 1:** Number of MRI studies and associated participant characteristics

	*N*	Age, years	Female (%)	Education, years	MMSE
CH	105	64.7 (6.1)	64	12.7 (3.2)	29.5 (0.6)
SCI	184	65.7 (7.1)	62	13.0 (3.7)	29.1 (1.1)†
MCI	249	67.0 (7.1)*	56	12.2 (3.7)	28.0 (1.6)†
Dementia	214	68.9 (6.8)†	55	11.3 (3.5)†	23.4 (4.5) †
*AD*	98	68.0 (6.6)†	62	11.1 (3.4)*	22.3 (5.2) †
*SVD*	25	69.6 (6.4)†	24†	11.2 (3.3)*	26.0 (1.9)†
*Mixed*	51	72.2 (5.8)†	69	11.2 (3.7)*	22.7 (4.1)†
*Other*	40	66.7 (7.3)	40*	11.6 (3.7)	25.0 (3.0)†

Data are given as mean (standard deviation (SD))

Data concerning education was missing in 32 cases, evenly spread in the SCI, MCI and dementia group. MMSE data was missing in 14 cases, all in the dementia subgroups except for the SVD group

*CH* Cognitively healthy, *SCI* Subjective cognitive impairment, *MCI* Mild cognitive impairment, *AD* Alzheimer’s disease, *SVD* Subcortical vascular dementia, *Mixed* Mixed dementia, *Other* Other dementia, *MMSE* Mini mental state examination

*Significant results compared to CH, *p* < 0.05. † Significant results compared to CH, *p* < 0.001

### Image acquisition

The MRI protocol performed as part of the Gothenburg MCI study included a T_1_-weighted MPRAGE 3D volume scan used for MTA scoring and volumetric measurements. Between years 1999 and 2004, MRIs were performed on a 0.5 Tesla MR scanner (Philips NT5, Eindhoven, The Netherlands). The following scan parameters were used: repetition time (TR) 30 ms; echo time (TE) 10 ms; slice thickness 1.5 mm; slice gap 0 mm; flip angle 40°; field–of-view (FOV) 220 × 220 mm^2^; acquisition pixel size 0.86 × 1.12 mm^2^; and reconstruction pixel size 0.86 × 0.86 mm^2^. Between years 2005 and 2014 participants were examined on a 1.5 Tesla MR scanner (Siemens Symphony, Siemens Medical Systems, Erlangen, Germany) (TR 1610 ms; TE 2.38 ms; slice thickness 1 mm; slice gap 1 mm; flip angle 15°; FOV 250 × 203 mm^2^; acquisition pixel size 1.0 × 1.0 mm^2^; reconstruction pixel size 0.49 × 0.49 mm^2^).

### Image analysis

The T_1_-weighted 3D MPRAGE MRI data were used for volumetric measurements and visual ratings. All raters were blinded to clinical information.

#### Visual assessment

**Fig. 1:**
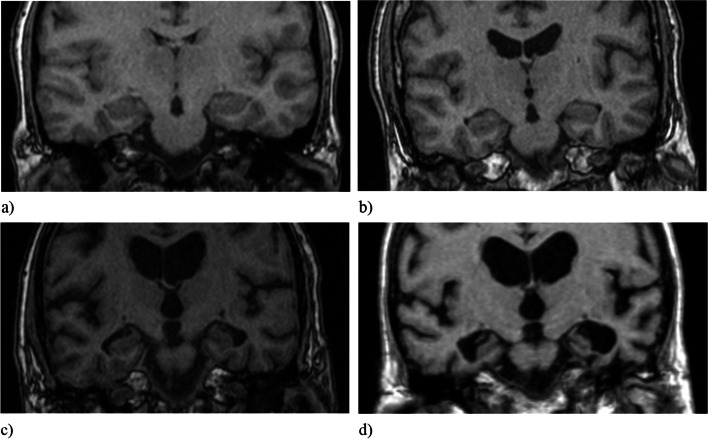
Coronal T_1_-weighted slices at the level of the hippocampus body of four different study participants with different levels of medial temporal lobe atrophy (MTA). **a** MTA 0 bilaterally. **b** MTA 1 bilaterally. **c** MTA 2 right side, MTA 3 left side. **d** MTA 4 bilaterally. Images (**a**-**c**) were acquired with a 1.5 Tesla scanner, whereas image (**d**) was acquired with a 0.5 Tesla scanner

Visual rating of MTA was performed within the Osirix software version 5.8.2 (Pixmeo, Geneva, Switzerland) viewing platform. The 3D T_1_-weighted data sets were reformatted in a coronal view, angulated perpendicularly to a line connecting the anterior and posterior commissure (AC-PC-line). Slabs of 3 mm thickness were reconstructed from the original 3D T_1_-weighted volume to increase signal to noise-ratio. The visual MTA rating was done separately for the right and left medial temporal lobe (MTL) in accordance to the method described by Scheltens et al. [17], i.e., it included the assessment of the hippocampal formation (hippocampus and para-hippocampal gyrus) and of the width of the surrounding cerebrospinal fluid (CSF) spaces, e.g. the temporal horn and the choroid fissure. The visual estimate of the volume of MTL structures results in subjective MTA scores ranging from 0 (no atrophy) to 4 (severe atrophy). In MTA 0, no CSF will be seen surrounding the hippocampus; in MTA 1, there is an increase of the width of the choroid fissure; in MTA 2–4, the temporal horn gradually enlarges and there is a gradual loss of height of the hippocampal formation (see Fig. 1).

MTA rating was performed by two raters, hereafter referred to as Rater 1 and Rater 2. Rater 1 received training by an experienced neuro-radiologist (Rater 2) including example rating and feedback for 100 data sets. Randomly selected subgroups were re-evaluated for both 0.5 Tesla MRI (*n* = 30) and 1.5 Tesla MRI (*n* = 74) by Rater 1 for intra-rater reliability calculations and by Rater 2 as second reader for inter-rater reliability calculations.

#### Volumetric assessment

Volumetric evaluation, previously performed on the same material for different studies, comprised assessment of the hippocampal volumes of 134 0.5 Tesla examinations using manual hippocampal volumetric measurement [14] and of 560 1.5 Tesla MRI examinations using the semi-automated software suite FreeSurfer version 5.3.0 as previously described [34].

### Statistical analysis

Demographical data were analyzed using independent-samples t-test for continuous data and χ square for nominal data. Group comparisons were performed using Mann–Whitney U test for MTA scores and independent-samples t-test for hippocampal volumes. Intra- and inter-rater reliability of MTA assessments was determined with Cohen’s weighted kappa statistics, which takes the ordered nature of the MTA scale into account. Linear correlation between ordinal MTA data and continuous hippocampal volume data was measured with a Spearman rank correlations test (ρ). In order to examine the group classification ability of mean MTA and hippocampal volumes with respect to specific dementia diagnoses, receiver operating characteristic (ROC) analysis was performed. Lastly, different MTA cut-off values were evaluated for the differentiation of participants with specific dementia diagnoses from cognitively healthy participants. Analyses were made separately for two age groups, in order to adjust for normal age-dependent hippocampal atrophy. Sensitivity and specificity for MTA cut-off points were calculated using cross tabulation. Statistical analyses were conducted in IBM SPSS, version 26 (IBM Corp., Armonk, N.Y., USA).

## Results

Participants with MCI or AD, SVD or mixed dementia, as shown in Table 1, were older than the cognitively healthy group. Fewer years of education were evident in the AD, SVD and mixed dementia groups than in CH. Compared to CH, mean MMSE scores were significantly lower in all other groups.

A box-and-whiskers plot of FreeSurfer hippocampal volume distributions identified 20 extreme outliers (> 3 × interquartile range (IQR)). In these cases, segmentations were of poor quality and reported volumes discrepant to visual assessment. Extreme outliers were hence deemed invalid and the volumes were excluded from further analyses.

### Reliability

For the 0.5 Tesla MRI exams, intra-rater weighted kappa values were 0.78 on both right and left sides. For the 1.5 Tesla exams, intra-rater weighted kappa was 0.71 on the right side and 0.80 on the left side. Inter-rater agreement for the 0.5 Tesla exams was 0.59 and 0.65 and for the 1.5 Tesla exams 0.53 and 0.67, on right and left side respectively.

### Correlation with quantitative hippocampal volumes

**Fig. 2:**
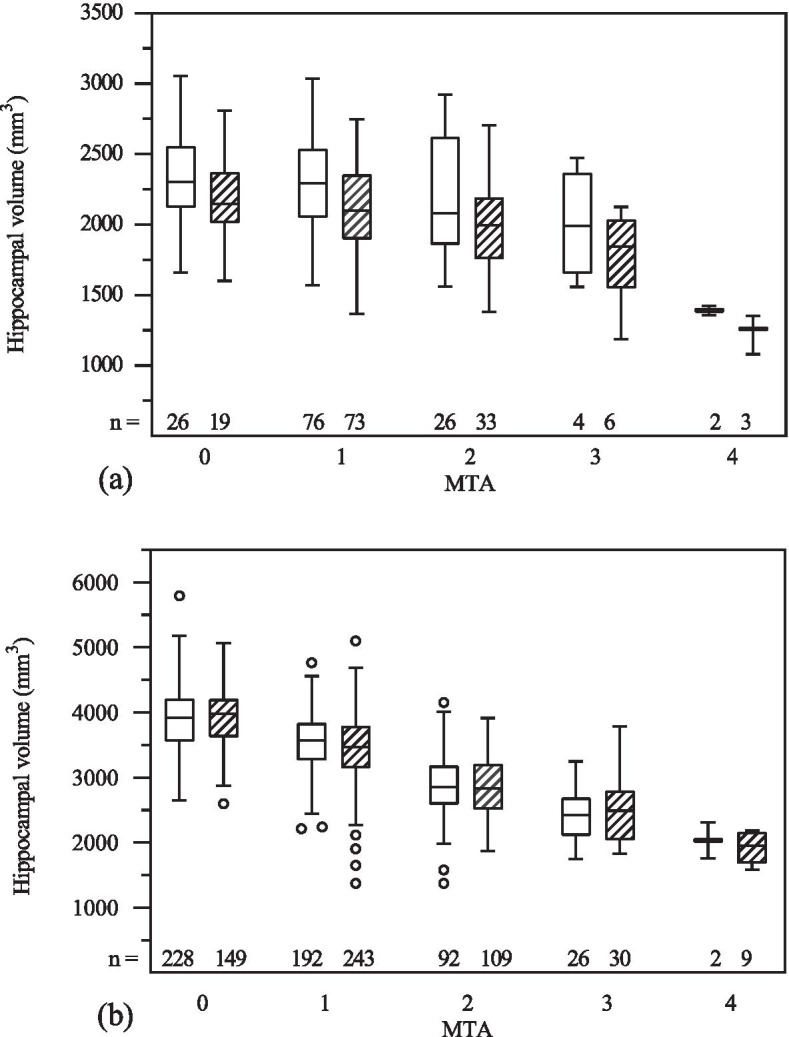
Tukey boxplot for hippocampal volume vs MTA determined with **a** manual volumetry and **b** FreeSurfer volumetry. Line inside box indicates median. Whiskers indicate ± 1.5 IQR (interquartile range). White boxes: right side. Hatched boxes: left side

Figure 2a and b illustrate hippocampal volumes in relation to MTA scores. The linear correlation between manually determined hippocampal volumes and MTA score was weak with a Spearman’s correlation coefficient of -0.20 (*p* < 0.05) on the right side and -0.31 (*p* < 0.001) on the left side. The linear relationship between FreeSurfer volume estimates and MTA score was moderate, with a Spearman’s correlation coefficient of -0.64 (*p* < 0.001) on the right side and -0.68 (*p* < 0.001) on the left side.

### Group differences

Mean MTA score was significantly higher and FreeSurfer volume significantly smaller in participants with SCI, MCI or any of the dementia subtypes than in cognitively healthy (CH) subjects (Table 2). Meanwhile, for manually determined hippocampal volumes a significant reduction compared to CH was only observed in AD and mixed dementia patients.

**Table 2:** Bilateral mean MTA score and hippocampal volume in patient groups

	*N*	Mean MTA score	*N*	Manual volumetry (mm^3^)	*N*	FreeSurfer volumetry (mm^3^)
CH	105	0.53 (0.46)	34	2201 (310)	57	3874 (475)
SCI	184	0.71 (0.63)*	6	2283 (393)	158	3699 (466)*
MCI	249	0.95 (0.76)†	63	2211 (360)	163	3477 (565)†
Dementia						
*AD*	98	1.71 (0.89)†	13	1954 (310)*	76	2871 (552)†
*SVD*	25	1.22 (0.68)†	6	2352 (199)	15	3477 (376)*
*Mixed*	51	1.64 (0.89)†	9	1936 (310)*	37	2809 (547)†

Data are given as mean of right and left sides (SD)

In participants with dementia other than AD, SVD or Mixed, manual volumetry was performed in three MRI exams and FreeSurfer volumetry in 34 MRI exams

*AD* Alzheimer’s disease, *CH* Cognitively healthy, *MCI* Mild cognitive impairment, *Mixed* Mixed dementia, *MTA* Medial temporal lobe atrophy, *SCI* Subjective cognitive impairment, *SVD* Subcortical vascular dementia

* Significant results compared to CH, *p* < 0.05. † Significant results compared to CH, *p* < 0.001

### Discrimination ability

The ability of mean MTA score and hippocampal volume to distinguish between patients with dementia subtypes and CH participants is reported in Table 3. Both MTA and FreeSurfer showed good discriminatory ability between AD and CH as well as between mixed dementia and CH. SVD was separated from CH to a fair degree by MTA and FreeSurfer, and not at all using manual volumetry.

**Table 3:** Discrimination ability of mean MTA score and hippocampal volumes between dementia subtypes and CH

	MTA	Manual volumetry	FreeSurfer volumetry
Dementia			
*AD*	0.87 (0.82–0.92)†	0.75 (0.60–0.91)*	0.91 (0.87–0.96)†
*SVD*	0.79 (0.68–0.90)†	0.36 (0.17–0.56)	0.75 (0.61–0.89)*
*Mixed*	0.86 (0.80–0.93)†	0.73 (0.56–0.91)*	0.94 (0.88–0.99)†

Data are given as Area under the curve, AUC (95% confidence interval (CI)), based on receiver operating characteristic analysis (ROC)

*AD* Alzheimer’s disease, *CH* Cognitively healthy, *Mixed* Mixed dementia, *MTA* Medial temporal lobe atrophy, *SVD* Subcortical vascular dementia

* *p* < 0.05, † *p* < 0.001

### MTA cut-off values

Table 4 provides age-range specific sensitivity and specificity percentages for different MTA score thresholds for the discrimination of investigated dementia entities from CH. For the age group below 75 years, at an MTA score threshold of 1, all three dementia subtypes were recognized with a sensitivity of over 80% (specificity 67.7%). In the age group ≥ 75 years, all CH (*n* = 6) were rated MTA ≥ 1. In this age group, most acceptable sensitivity and specificity resulted with a higher MTA threshold of 1.5. The SVD group at or above 75 years age is considered too small (*n* = 6) to provide reliable threshold values.

**Table 4:** Sensitivity and specificity (%) vs CH for different MTA score thresholds

MTA cut-off	Sensitivity			Specificity
	*AD*	*SVD*	*Mixed*	
≤ *74 years*				
*N*	77	19	26	99
0.5	96.1	89.5	92.3	36.4
1.0	81.8	84.2	80.8	67.7
1.5	66.2	47.4	53.8	97.0
2.0	48.1	26.3	42.3	100
≥ *75 years*				
*N*	21	6	25	6
0.5	95.2	83.3	100	0
1.0	90.5	66.7	88.0	16.7
1.5	76.2	50.0	72.0	66.7
2.0	61.9	16.7	48.0	100

MTA cut-off indicates lowest pathological mean MTA score (average right + left)

Dementia subtypes other than AD, vascular dementia and mixed dementia not shown (*n* (≤ 74 years) = 33, *n* (≥ 75 years) = 7)

*AD* Alzheimer’s disease, *CH* Cognitively healthy, *Mixed* Mixed dementia, *MTA* Medial temporal lobe atrophy, *SVD* Subcortical vascular dementia

## Discussion

Our objective was to examine reliability and validity of MTA in a memory clinic patient population. Intra and inter-rater agreement as a measure of reliability was found to be substantial to moderate. Validity of MTA was tested both with respect to correlation between MTA and quantitative hippocampal volumes and with respect to the ability of MTA to discriminate between dementia groups and CH. The MTA score correlated significantly with hippocampal volumes, and could readily separate AD and mixed dementia from the cognitively healthy group.

Intra-rater agreement was substantial, as interpreted according to Landis and Koch [35]. There was moderate to substantial inter-rater agreement, without any obvious difference between 0.5 Tesla and 1.5 Tesla images. Rater 2 showed a tendency to give higher scores than Rater 1, but out of a total of 208 ratings, comprising right and left side ratings of 104 MRI examinations, only two ratings differed more than one score point between the two raters. Inter-rater variability of the MTA scale has also been investigated in previous studies, with agreement varying from fair to good, with kappa values ranging from 0.28 to 0.51, up to a substantial agreement with a weighted kappa 0.84 [36–39]. A decrease in agreement over time for radiologists not working together has been shown [37]. In our case, Rater 1 was a radiology resident and Rater 2 an experienced neuro-radiologist working in a different department. The level of expertise of the raters might influence the rating, although while one study that compared expert with non-expert readers observed improved performance with extended practise in non-expert readers [40], another study found no difference in inter-rater agreement due to level of experience [36].

Validity was assessed in two ways: a) as correlation between MTA and quantitative hippocampal volumes and b) as the ability of the MTA score to discriminate among patient groups. The correlation between FreeSurfer hippocampal volumes and MTA was moderate, but a weaker correlation, yet still statistically significant, was observed for manual volumetry. Our results, based on a heterogeneous study population, are in line with previous studies, with similar modest correlations between manual volumetry and MTA [22–24], and higher correlations in studies using (semi-)automated methods, such as FreeSurfer or NeuroQuant [25, 26]. Despite such findings, good agreement of hippocampal volumes has been reported between FreeSurfer and manual volumetry [41, 42], although different definitions of anatomical boundaries lead to a bias with larger FreeSurfer volumes than manually determined volumes [43].

Both MTA score and FreeSurfer volumes permitted good discrimination between the AD group and CH group, with AUC values comparable to previous studies [19, 26, 44, 45]. Based on MTA score and FreeSurfer volumes, good discrimination between mixed dementia patients and CH group was also attained. As can be expected, considering the underlying neurodegeneration, the mixed dementia group showed increased MTA scores and decreased hippocampal volumes to almost the same extent as the AD group. Patients with SVD had also higher MTA scores and smaller FreeSurfer hippocampal volumes than the CH group, supporting previous reports of concurrent hippocampal atrophy in SVD [4–6, 8]. Although FreeSurfer volumes of patients with MCI and SVD were almost indistinguishable, MTA scores were higher in the SVD group (*p* < 0.05). This finding may reflect that the MTA score not only assesses hippocampal volume but also the surrounding CSF spaces, which might be indicative of subcortical and global brain atrophy [24, 46], rather than isolated hippocampal atrophy. Whereas subcortical atrophy may be a feature of SVD, the MCI group is heterogeneous and contains participants who remain cognitively stable.

MTA cut-off values that differentiate patients with AD from controls have previously been suggested by different research groups, and range from ≥ 1 to ≥ 2.5 depending on patient age [44, 47, 48]. In the present material, recommended threshold values are MTA ≥ 1 in the age group below 75 years and MTA ≥ 1.5 in participants 75 years or older. In contrast with previous studies, we tested the various cut-off values in SVD and mixed dementia groups as well as in AD, and found similar sensitivity for SVD in the younger age group and mixed dementia as for AD.

We have selected cut-off values that prioritize sensitivity over specificity levels. Higher cut-off levels, of MTA ≥ 1.5 and MTA ≥ 2, respectively, could be justified to avoid false positive tests, but at the cost of a lower detection rate. With the proposed thresholds, 31 out of 149 examinations of participants with confirmed AD or mixed dementia would have been classified as having no MTL atrophy. FreeSurfer hippocampal volumes were available in 23 of these “misclassified” examinations. Comparison of their mean FreeSurfer volumes showed a significantly larger volume in the misclassified group, with 3479 (SD 417) mm^3^ vs 2690 (SD 457) mm^3^ in the correctly classified group (*p* < 0.001), suggesting that the MTA scores reflect actual hippocampal size and as previously reported [49] there may indeed be a subset of AD patients without pronounced hippocampal atrophy. The variation of proposed cut-off values in studies may naturally also be affected by the subjective nature of the MTA scale. A smaller study reported different optimal cut-off values set by the two raters [50], even though inter-rater correlations were high. The accuracy of the MTA cut-off increased when the average between the two raters were used. Consensus decision of several raters was applied in the original study of MTA [17], which, however, is seldom practicable in routine clinical work.

The present study suggests that MTA is a reliable and valid marker of MTL atrophy even in a heterogeneous patient population. MTL atrophy is not specific to AD and our findings indicate that MTA is sensitive to atrophy also in patients with SVD and mixed dementia. As MTA is associated with cognitive dysfunction in patients with cerebral vascular disease as well as in AD, MTA is an important piece of information that should be reported and should be regarded along with other radiological findings in patients with cognitive impairment.

Limitations of our study include the transition between two MRI scanners operating at different field strength, reflecting the reality in many radiology departments, where the installed MRI systems often consist of scanners from different manufacturers and of different field strengths. For the purposes of this study, MTA ratings from 0.5 Tesla and 1.5 Tesla MRI exams were not distinguished in the statistical analysis. Eventual influence of field strength on the correlation assessment between MTA ratings and volumetric methods was not accessible, since manual volumetry was performed only on 0.5 Tesla scans and FreeSurfer volumetry only on 1.5 Tesla scans. To best of our knowledge, no previous studies have compared MTA performance at different field strengths. One study [51], however, reported substantial to excellent agreement between 1.5 Tesla MRI and 64-detector row computed tomography (CT) images, a modality which offers clearly less image contrast than 0.5 Tesla MRI. Another limitation is the small group sizes in the older age group. This was particularly notable when testing MTA cut-off points, where specificity values should be interpreted with caution. Few examinations were assigned the highest MTA score, possibly affecting linear correlations.

## Conclusions

In conclusion, our findings suggest that the MTA scale is a reliable and valid marker of medial temporal lobe atrophy and of use in the assessment of patients with cognitive impairment, even in a heterogeneous clinical patient population.

## Data Availability

The datasets generated and/or analysed during the current study are not publicly available due to privacy of study participants but are available from the corresponding author on reasonable request.

## Abbreviations

AC-PC: Anterior and posterior commissure
AD: Alzheimer’s disease
AUC: Area under the curve
CDR: Clinical Dementia Rating
CH: Cognitively healthy
CI: Confidence interval
CSF: Cerebrospinal fluid
CT: Computed tomography
EXIT: Executive Interview
FOV: Field–of-view
GDS: Global deterioration scale
IQR: Interquartile range
MCI: Mild cognitive impairment
Mixed: Mixed dementia
MMSE: Mini mental state examination
MRI: Magnetic resonance imaging
MTA: Medial temporal lobe atrophy
MTL: Medial temporal lobe
ROC: Receiver operating characteristic
SCI: Subjective cognitive impairment
SD: Standard deviation
STEP: Stepwise Comparative Status Analysis
SVD: Subcortical vascular dementia
TE: Echo time
TR: Repetition time
WMC: White matter changes
